# Supplementary material for: Impact of fabrication errors and refractive index on multilevel diffractive lens performance
Source: Sci Rep. 2020 Sep 3;10:14608. doi: 10.1038/s41598-020-71480-2 (PMC7471910; doi:10.1038/s41598-020-71480-2)
Supplement: Supplementary file 1 — Supplementary information. [file 41598_2020_71480_MOESM1_ESM.docx]

**Supplementary Information**

**Impact of Fabrication Errors and Refractive Index on Multilevel Diffractive Lens Performance**

Sourangsu Banerji^1^, Jacqueline Cooke^1^ and Berardi Sensale-Rodriguez^1, *^

^1^ Department of Electrical and Computer Engineering, The University of Utah, Salt Lake City, UT 84112, USA

* E-mail: [berardi.sensale@utah.edu](mailto:berardi.sensale@utah.edu)

1. **Refractive index and absorption coefficient of PLA**

**
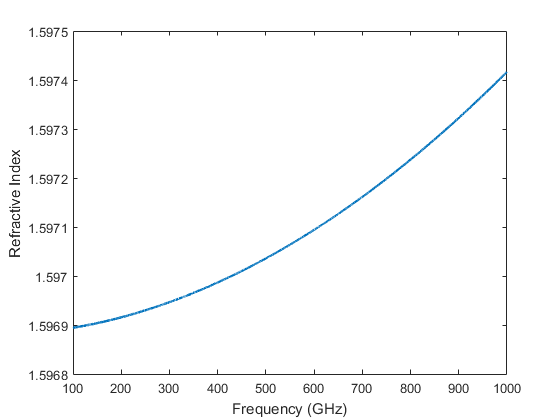
**

**Fig. S1.** Refractive Index of PLA polymer

**
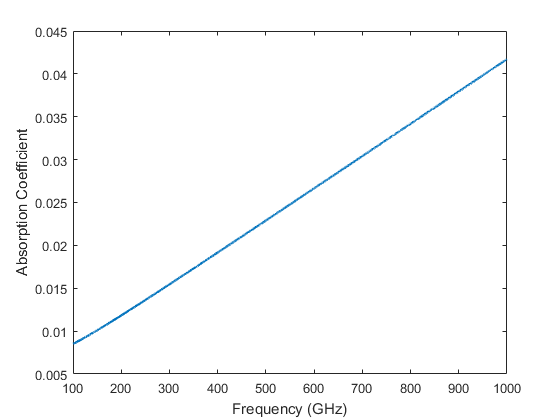
**

**Fig. S2.** Absorption co-efficient of PLA polymer

1. **MDL designs**

**
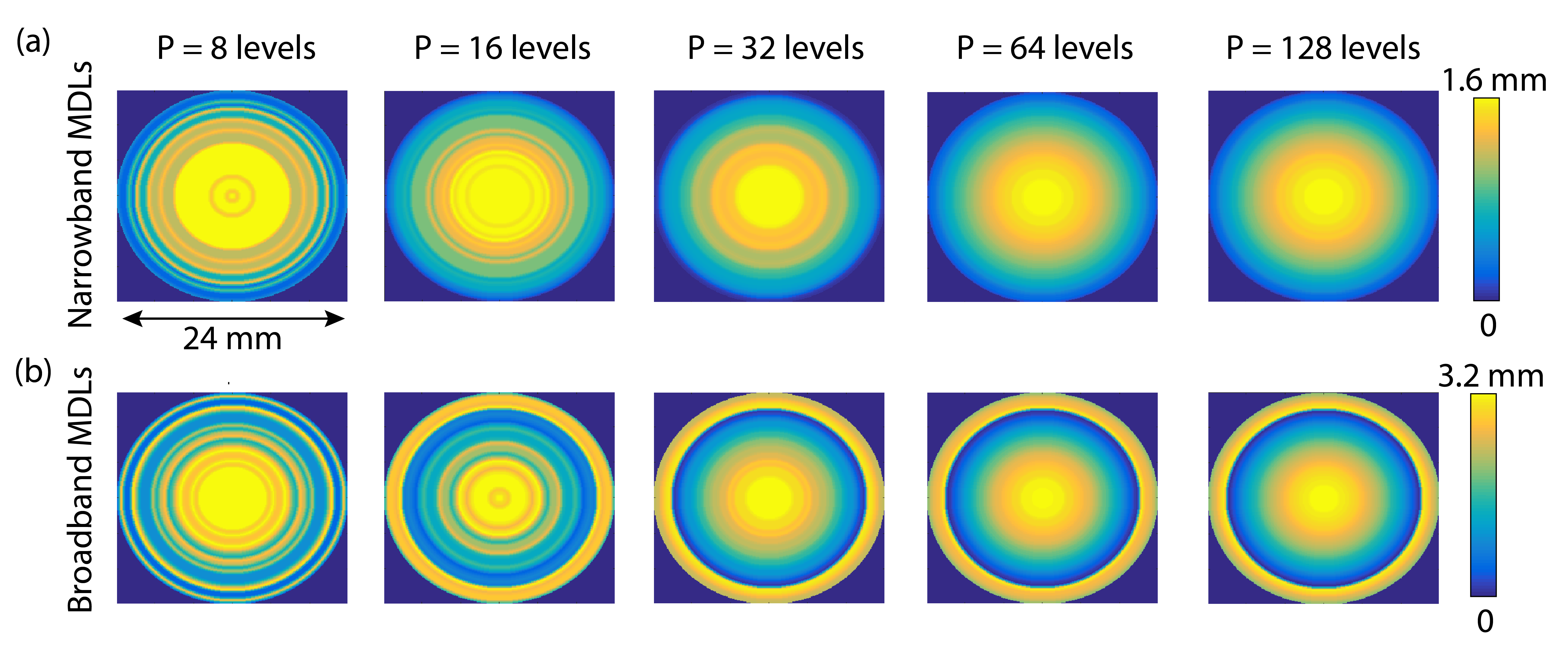
**

**Fig. S3.** MDL designs for P = [8, 16, 32, 64, 128] for **(a)** narrowband and **(b)** broadband

1. **Additional MDL designs**

**
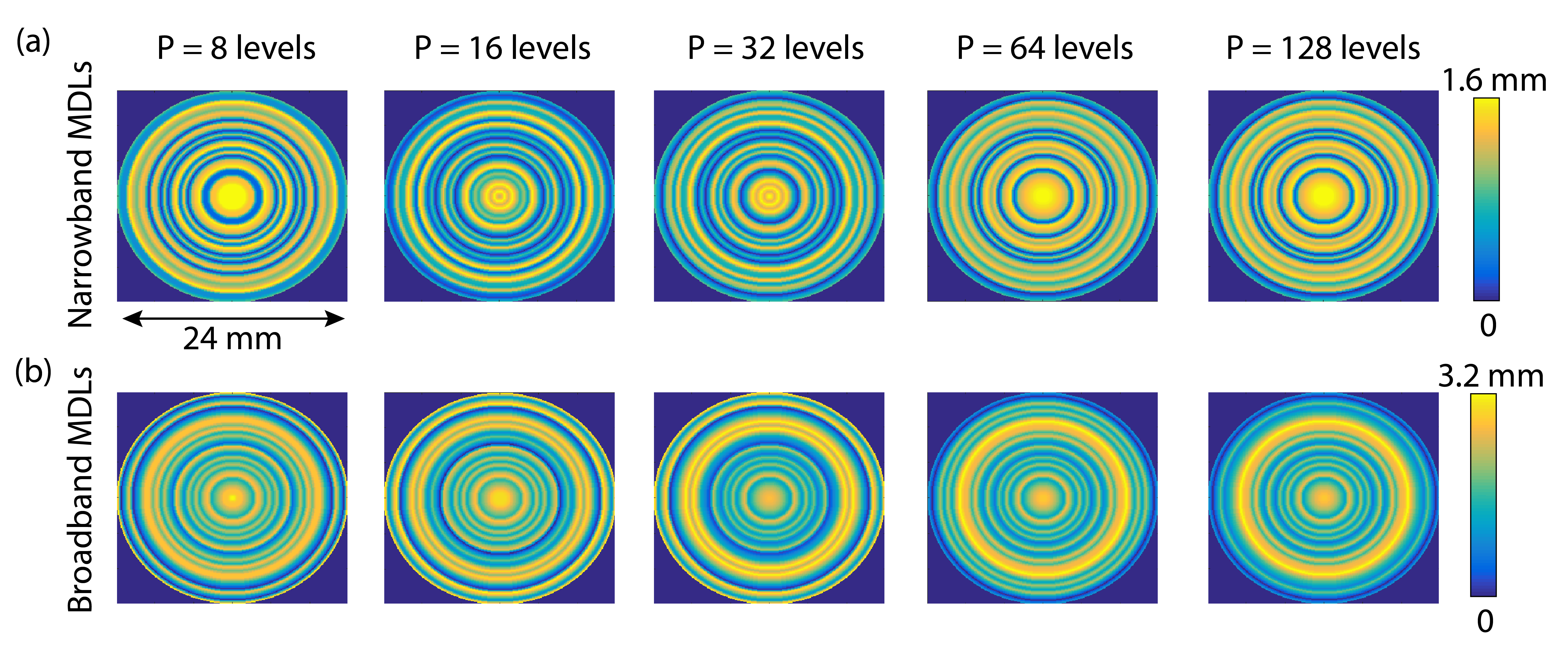
**

**Fig. S4.** Additional MDL designs for P = [8, 16, 32, 64, 128] for **(a)** narrowband and **(b)** broadband

1. **Effect on average focusing efficiency due to a standard deviation-based error in ring height and ring width for the additional MDL designs**

**
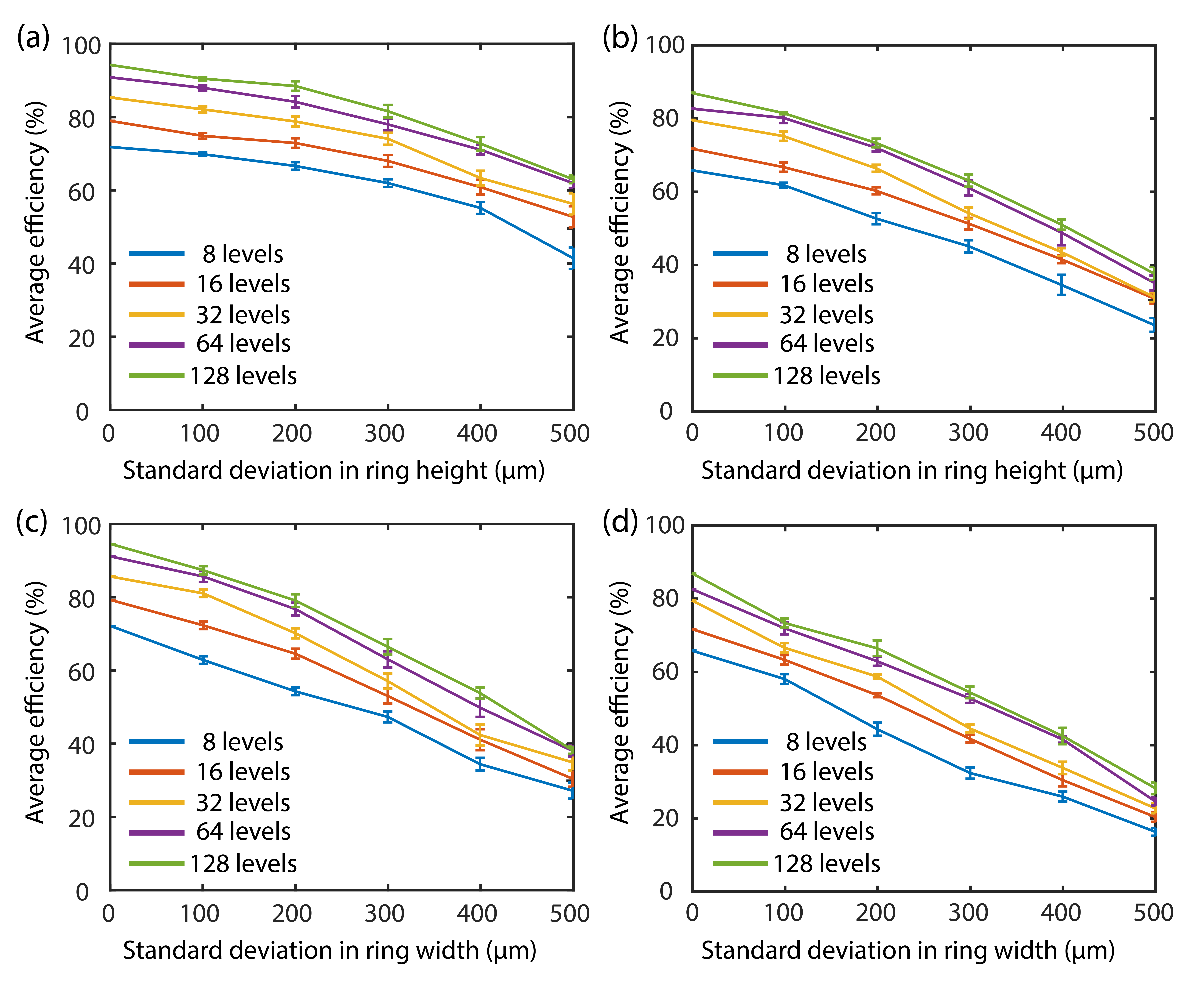
**

**Fig. S5.** Effect on average focusing efficiency due to a standard deviation-based error in ring height for (***a***) narrowband operation at 0.2 THz and (***b***) broadband operation from 0.1 THz to 0.3 THz. A similar approach is undertaken to characterize the impact due to a standard deviation-based error in ring width under (***c***) narrowband operation at 0.2 THz and (***d***) broadband operation from 0.1 THz to 0.3 THz.

1. **Impact on average efficiency due to a standard deviation-based error in ring height for a fixed error in ring width and vice versa for the additional MDL designs**

**
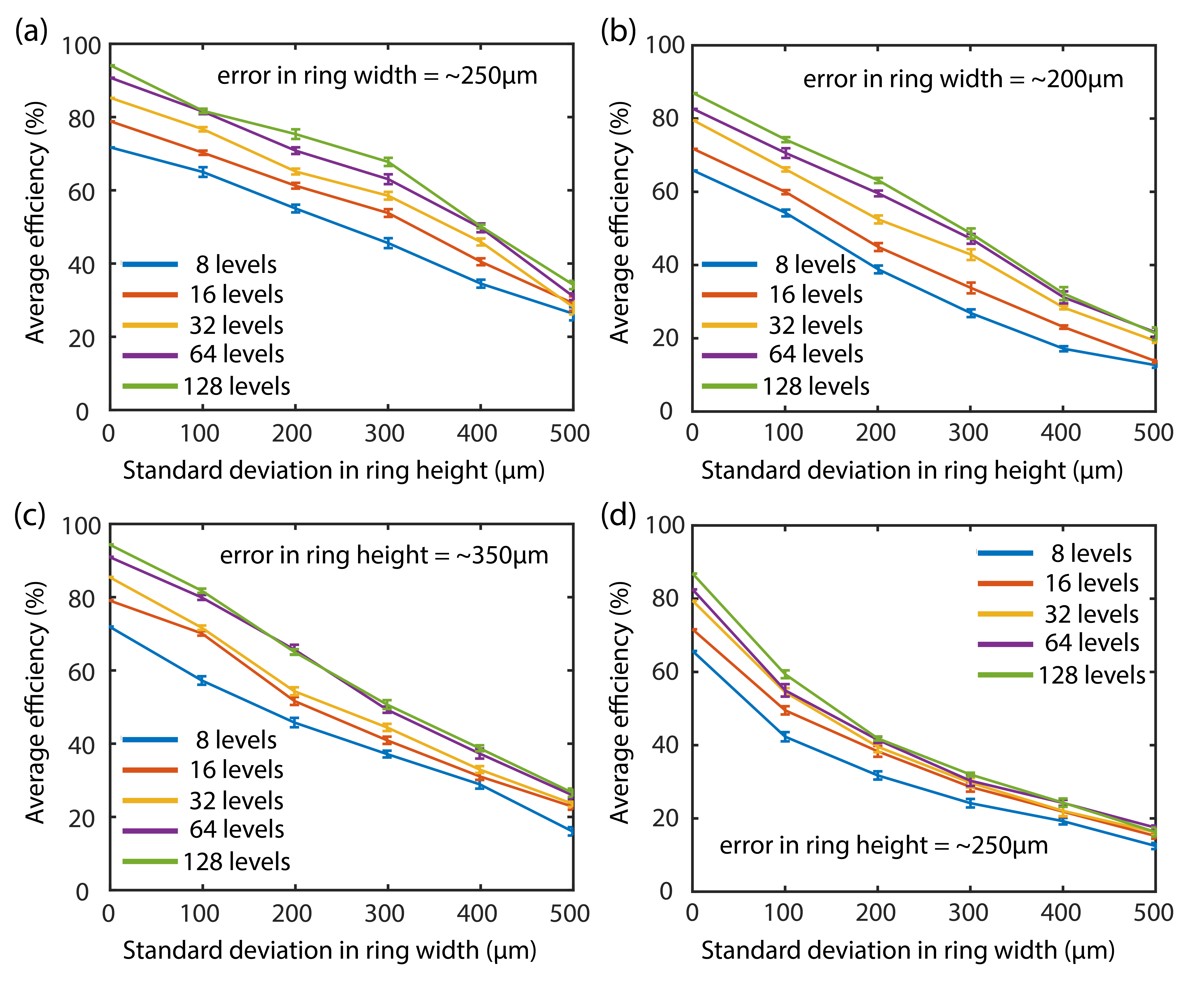
**

**Fig. S6.** Impact on average efficiency due to a standard deviation-based error in ring height for a fixed error in (***a***) width = ~250 µm under narrowband operation at 0.2 THz and (***b***) width = ~200 µm under broadband operation from 0.1 THz to 0.3 THz. Consequently, the impact due to a standard deviation-based error in ring width for a fixed error in (***c***) height = ~350 µm under narrowband operation at 0.2 THz and (***d***) height = ~250 µm under broadband operation from 0.1 THz to 0.3 THz.

1. **Exemplary PSFs for the narrowband MDL design with P = 128 at f = 0.2 THz**

**
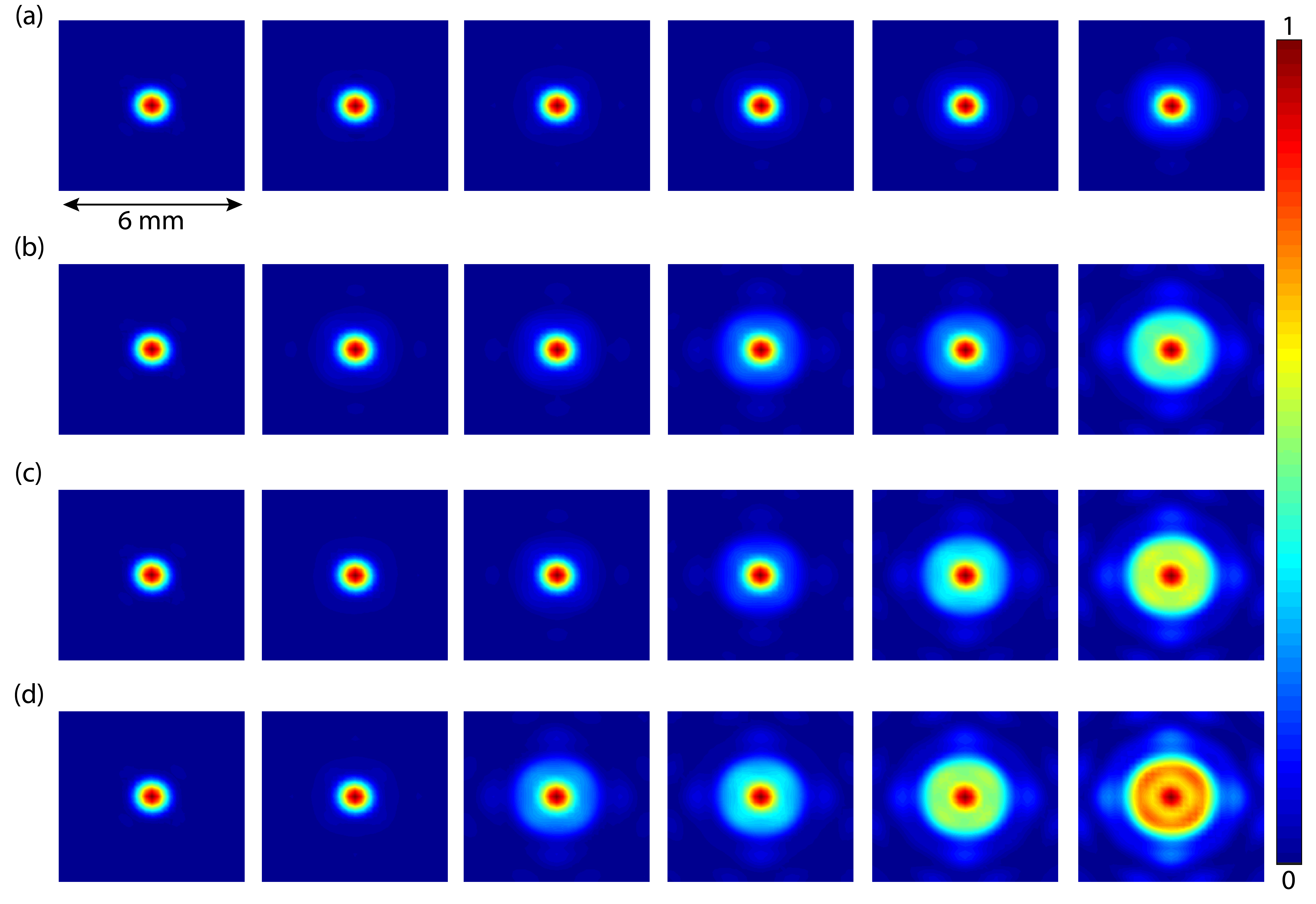
**

**Fig. S7.** Exemplary PSFs for the MDL under narrowband operation at 0.2 THz with the number of pixel height level (P) = 128 due to a standard deviation-based error in (***a***) ring height and (***b***) ring width. Consequently, the exemplary PSFs for the same MDL due to a standard deviation-based error in (***c***) ring height for a fixed error in width = ~250 µm and (***d***) ring width for a fixed error in height = ~350 µm.

1. **Localized effects arising from errors in the inner (central) and outer (periphery) rings of the MDL**


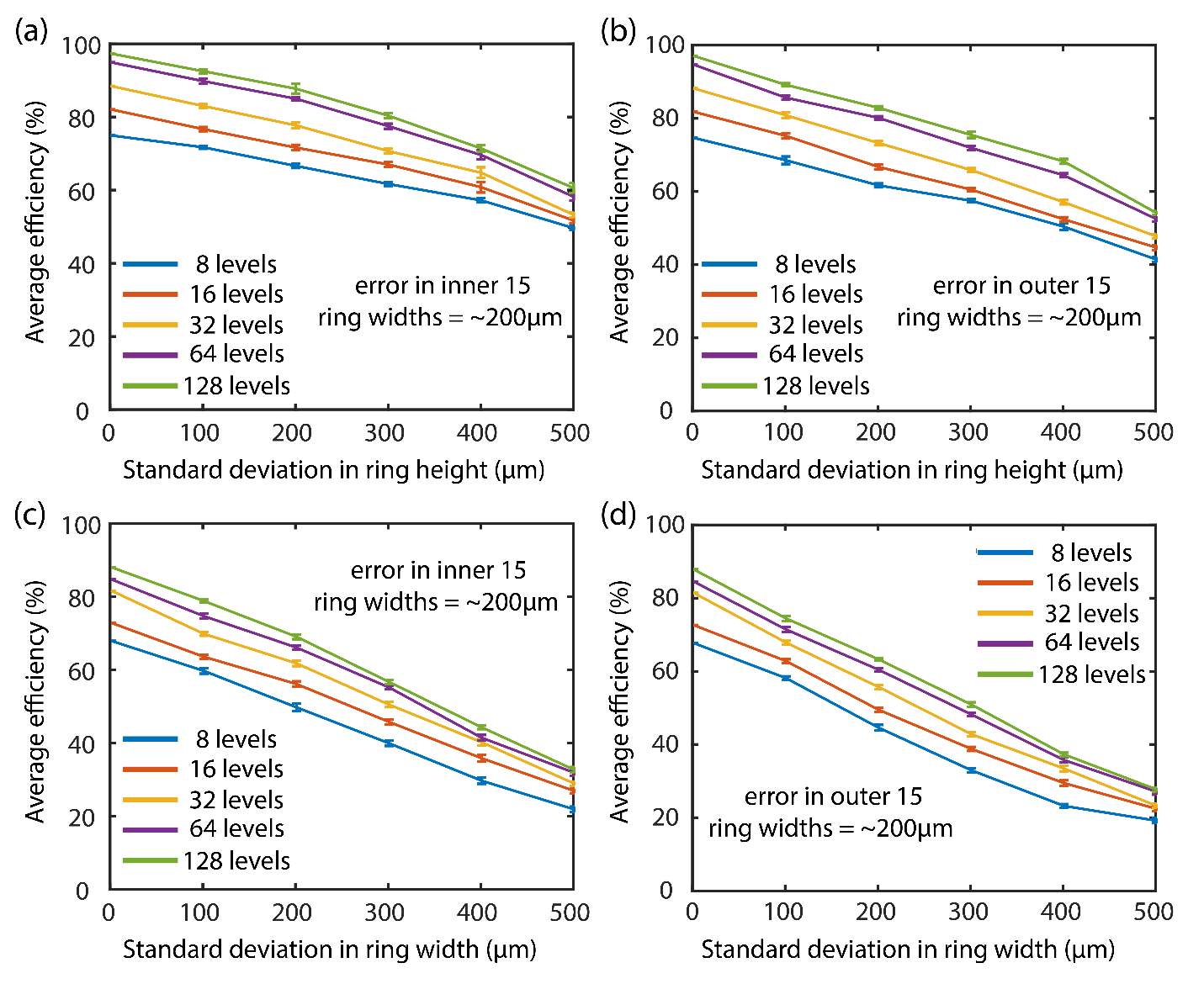


**Fig. S8.** Impact on average efficiency due to a standard deviation-based error in ring height under narrowband operation at 0.2 THz for a fixed error in width = 200 µm in the (*a*) inner 15 MDL rings (center) and (*b*) outer 15 MDL rings (periphery). Consequently, the impact on average efficiency due to a standard deviation-based error in ring height under broadband operation from 0.1 THz to 0.3 THz for a fixed error in width = 200 µm in the (*c*) inner 15 MDL rings (center) and (*d*) outer 15 MDL rings (periphery).
